# Supplementary material for: Characterization of Calpain and Caspase-6-Generated Glial Fibrillary Acidic Protein Breakdown Products Following Traumatic Brain Injury and Astroglial Cell Injury
Source: Int J Mol Sci. 2022 Aug 11;23(16):8960. doi: 10.3390/ijms23168960 (PMC9409281; doi:10.3390/ijms23168960)
Supplement: Supplementary file 1 [file ijms-23-08960-s001.zip › ijms-1803035-supplementary.pdf]

IJMS Ms Supplementary Materials Yang et al.

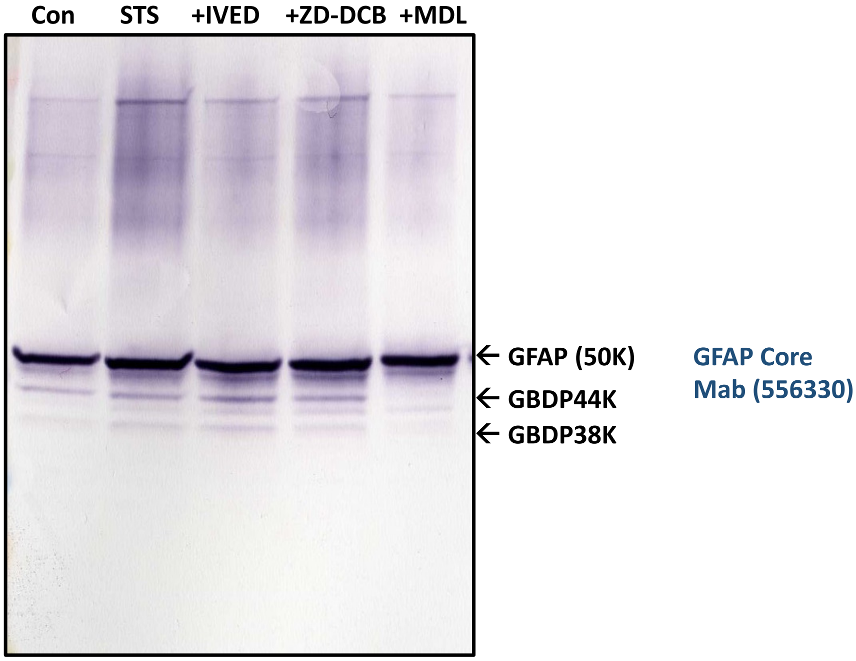

Figure S1. Effects of caspase-6 and calpain inhibitor on GBDP-38K production in rat cerebrocortical culture after STS treatment. Data shown is representative of N=3. STS; staurosporine (1 μM for 24 h) in the presence or absence of calpain inhibitor MDL-28170 (50 μM), caspase-6 inhibitor Z-IVED-FMK (50 μM) or pan-caspase inhibitor Z-D-DCB. STS generated GBDP38K was not blocked by Z-IVED-FMK or Z-d-DCB, but by MDL28170.

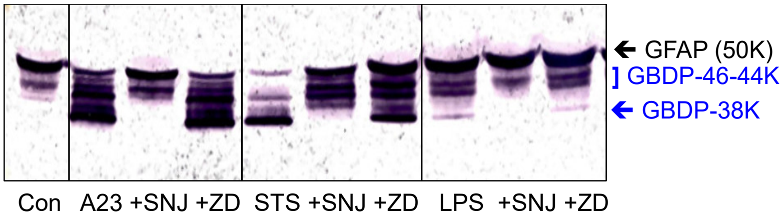

Figure S2. GFAP proteolysis in rat primary glial culture after cytotoxin-challenges as well as LPS (200 ng/mL) stimulation for 24 h. Data shown is representative of N=3. A232: A23187, STS; staurosporine, SNJ; calpain inhibitor SNJ1945, Z_D- pan caspase inhibitor Z-D-DCB

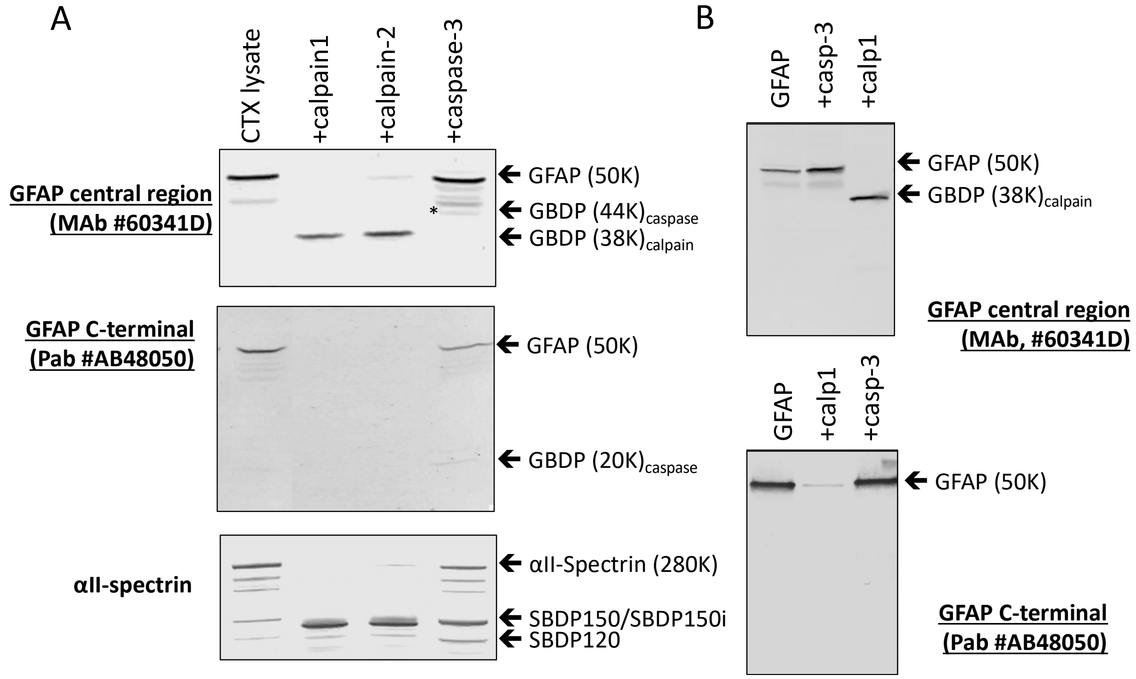

Figure S3. GFAP is relatively resistant to caspase-3 digestion. (A) GFAP from cortical mixed culture lysate is relatively resistant to in vitro digestion by caspase-3 generating 0nly minor appearance of 44K and 20K fragments. (B) Purified recombinant GFAP is also resistant to caspase-3 digestion.

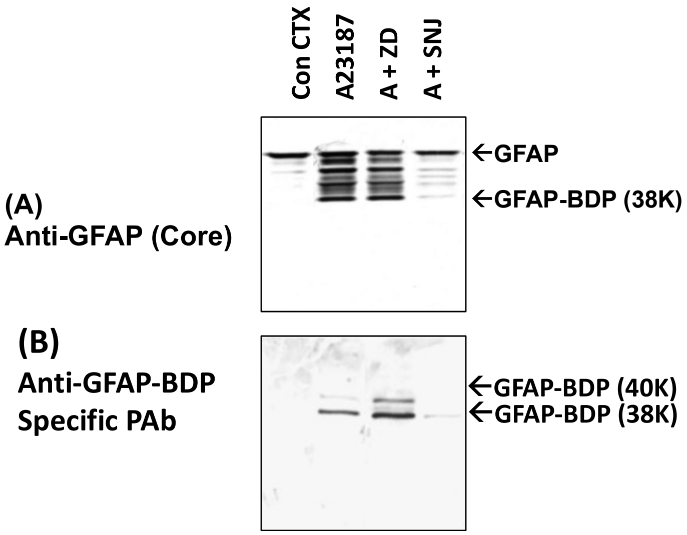

Figure S4. **Full length human GFAP digestion by calpain generated several C-terminal LMW peptides. GBDP-specific antibody detected GFAP-fragments of 40k, 38K were specifically sensitive to calpain inhibitor SNJ-1945, but not sensitive to pan caspase inhibitor Z-D-DCB. (A) anti-GFAP-Core Ab, (B) GBDP-N-term PAb.**

Table S1. Severe TBI patient demographic data.

Number		21
Age, mean +/- SD		32+/-10
Gender, n	Male	17
	female	4
Motor Glascow Coma Score, median/range		4/1-6
Pupils, n	Both reactive	13
	One reactive	1
	Neither reactive	7
Marshall CT category, n	Diffuse injury 1	0
	Diffuse injury 2	5
	Diffuse injury 3	5
	Diffuse injury 4	3
	Evacuated mas	8
	Unevacuated mas	0
Mechanism of Injury	Motor vehicle accident	13
	Fall	1
	Assault	6
	Other	1
6 month Glascow Outcome Scale, n	Good recovery	1
	Moderate disability	6
	Severe disability	8
	Vegetative	1
	Dea	3
	Lost to follow up	2
6 Month Disability Rating Scale, median/range		6/0-30

Table S2. GFAP peptides LC-selected reaction monitoring (SRM)- MS/MS parameters

Compound	Start time (min)	End time (min)	Precursor (min)	Product (m/z)	Collision energy	Min Dwell time (ms)
DGEVIKE (+2)	0	50	395.203	276.155	17.8	9.957
DGEVIKE (+2)	0	50	395.203	389.239	11.8	9.957
DGEVIKE (+2)	0	50	395.203	488.308	13.8	9.957
DGEVIKE (+2)	0	50	395.203	617.35	11.8	9.957
DGEVIKE (+2)	0	50	395.203	674.372	15.8	9.957
DGEVIKE (heavy) (+2)	0	50	398.209	276.155	17.8	9.957
DGEVIKE (heavy) (+2)	0	50	398.209	389.239	11.8	9.957
DGEVIKE (heavy) (+2)	0	50	398.209	494.322	13.8	9.957
DGEVIKE (heavy) (+2)	0	50	398.209	623.364	11.8	9.957
DGEVIKE (heavy) (+2)	0	50	398.209	680.386	15.8	9.957
DGEVIKES (+2)	0	50	438.719	235.092	13.2	9.957
DGEVIKES (+2)	0	50	438.719	363.187	15.2	9.957
DGEVIKES (+2)	0	50	438.719	476.271	13.2	9.957
DGEVIKES (+2)	0	50	438.719	575.34	13.2	9.957
DGEVIKES (+2)	0	50	438.719	704.382	13.2	9.957
DGEVIKESb(+2)	0	50	438.719	761.404	15.2	9.957
DGEVIKES (heavy)(+2)	0	50	441.725	235.092	13.2	9.957
DGEVIKES (heavy)(+2)	0	50	441.725	363.187	15.2	9.957
DGEVIKES (heavy)(+2)	0	50	441.725	476.271	13.2	9.957
DGEVIKES (heavy)(+2)	0	50	441.725	581.354	13.2	9.957
DGEVIKES (heavy)(+2)	0	50	441.725	710.396	13.2	9.957
DGEVIKES (heavy)(+2)	0	50	441.725	767.418	15.2	9.957
